# How Relevant is Food Craving to Obesity and Its Treatment?

**DOI:** 10.3389/fpsyt.2014.00164

**Published:** 2014-11-19

**Authors:** Marc N. Potenza, Carlos M. Grilo

**Affiliations:** ^1^Departments of Psychiatry, Neurobiology and Child Study Center, Yale University School of Medicine, New Haven, CT, USA; ^2^Department of Psychiatry and Psychology, Yale University, New Haven, CT, USA

**Keywords:** food addiction, food craving, naltrexone, bupropion, obesity, binge-eating disorder

Cravings represent strong motivational states that are characterized by intense desires typically relating to the anticipation of consuming pleasure-producing substances or engaging in hedonic behaviors. In considering food craving and the extent of its applicability to food, a brief review of the history of craving within a culture-sensitive framework appears warranted. Many cultures appear to have considered cravings in different contexts over time, although it has been contended, based on analyses of translations and lexicalization across languages, that craving may fail to translate outside of Europe and North America, although there are similarities in the use of craving and addiction across domains of use (1). The word “crave” is derived from the Old English crafian meaning to beg^1^. Over time, the term craving became linked to excessive patterns of substance use. For example, in the early nineteenth century, in conceptualizing excessive patterns of alcohol consumption, the term dipsomania (translated from the German term *Trunksucht*, or drinking addiction) was described to define alcoholism as a condition characterized by a craving for continued intoxication (2). In Buddhism, the term tan_._hā is commonly translated to mean craving (although its literal translation is “thirst”), with kāmatan_._hā (sense-craving) describing strong motivations to experience pleasant feelings or sensory pleasures^2^. In Buddhism, tan_._hā is seen as a type of ignorant desire and a cause of suffering and negative affective states, and some current approaches to understanding treatment mechanisms and promoting treatment development in addictions have involved considering craving within a Buddhist context (3, 4). Thus, links between cravings and negative processes including addictions have a longstanding history across multiple cultures.

In current psychiatric conceptualizations of addictions, cravings are considered an important component. Although substance-use disorders have been included in prior editions of the Diagnostic and Statistical Manual, a change from DSM-IV to DSM-5 involved the addition of an inclusionary criterion targeting craving in the diagnosing of substance-use disorders (5, 6). Despite the only recent addition of craving to the formal diagnostic criteria for substance-use disorders, craving has long been considered an important and clinically relevant feature of substance-use disorders. Craving has, for example, been linked in important fashions to treatment outcomes for both pharmacological interventions [e.g., naltrexone in the treatment of alcohol dependence (7)] and behavioral therapies [e.g., cognitive-behavioral therapies (8)] for substance addictions. Findings linking craving and treatment outcomes also appear applicable to non-substance or behavioral addictions; for example, in individuals with pathological gambling receiving opioid-receptor antagonists (naltrexone or nalmefene), individuals with strong gambling urges or cravings at treatment onset were more likely to demonstrate a better treatment outcome (9).

Despite the apparently widely appreciated relevance of craving to substance-use disorders and their treatment, the relevance of addiction features, including craving, to eating behaviors and conditions relating to excessive eating [e.g., obesity or binge-eating disorder (BED)] is more controversial and a topic of considerable debate (10–13). Some investigators have posited that energy balance remains central to obesity and that addiction or related aspects may represent a relatively minor component (13). Other investigators have suggested that a rapidly changing food environment may be contributing to the increases in obesity that have been observed over the past 30–40 years (14). Specifically, given the relative abundance and availability of inexpensive foods, it is possible that motivations to consume highly palatable foods, and perhaps large portions thereof, have taken a larger role in contributing to eating behaviors than in years past when the motivation to eat may have been more closely linked to energy restoration (15). Thus, examining other addiction-related constructs, such as food craving, as they relate to obesity and other food-related conditions seems relevant.

Multiple and diverse studies suggest that food cravings may be clinically relevant to understanding aspects of obesity and associated forms of disordered eating such as BED. Naturalistically and clinically, many individuals with overeating concerns and with BED report seeking and attending groups such as Overeaters Anonymous and other addiction-based 12-step programs (16). Researchers have developed specific measures to assess food addiction constructs [e.g., the Yale Food Addiction Scale, which has been investigated and validated to varying degrees across different clinical, age, racial, and cultural groups (17–22)] and, more specifically, various models and aspects of “food craving” (23–25) in order to investigate relationship with clinically relevant measures. For example, food craving has been linked to body mass index and consumption of multiple types of foods (sweet, high-fat, carbohydrate/starches, and fast-food) in community-dwelling individuals (26) and to various non-clinical and clinical study groups of individuals following dietary restrictions (27–29). Food cravings may also discriminate between successful and unsuccessful dieters (30, 31). Environmental factors like stress may induce food cravings and influence eating behaviors (32), and such effects may be particularly relevant to women (33, 34).

Importantly, relationships between food cravings and clinically relevant measures may differ in specific groups (25). For example, studies have reported significant differences in food cravings and associated clinical features between obese persons with and without BED (24, 25, 35, 36). As expected, individuals who endorse “food addiction” symptoms also report higher food cravings (37). Consistent with some research suggesting similarities in craving across different consummatory behaviors and addictions (38), research has found similarities in food cravings between women with obesity and women who smoke tobacco (39) and higher frequencies of substance-use disorders among obese women with BED who smoke than do not smoke (40).

Relationships between food cravings and various biological variables perhaps differing across specific groups have also been reported. For example, food-craving responses to favorite-food cues were associated with measures of insulin resistance in individuals with obesity but not in those of lean body mass, with thalamic brain activation mediating this relationship in the group with obesity (41). These findings suggest a biological mechanism linking insulin resistance and food cravings in obesity that might involve the thalamus, a region shown to differ in obese and lean humans in norepinephrine transporter availability (42). As such it is tempting to speculate that drugs targeting noradrenergic systems might be helpful in targeting food cravings in obesity, although this remains speculative and warrants further investigation. However, other systems [e.g., involving dopamine release (43)] appear differentially linked to food craving in obesity, suggesting contributions from multiple biological systems to food cravings. Additional, non-mutually exclusive pathways appear differentially linked to food craving and regional brain activations in obese and non-obese individuals. For example, the naturally occurring satiety lipid oleoylethanolamide appears differentially linked to body-mass-index measures in obese and lean individuals and to show different relationships with insular activations in response to food cues (44). Furthermore, molecular entities linked to appetite regulation and body habitus (e.g., leptin, ghrelin) appear differentially associated with regional brain activations to food cues in obese versus non-obese individuals and implicated in substance-use disorders (45, 46). These findings raise the possibility that common mechanisms may underlie craving states in obesity and substance-use disorders. Consistent with this possibility, meta-analyses of brain imaging data suggest common contributions of multiple brain regions to drug and food cravings (47). These commonalities have implications for treatment development in that treatments may be applicable to multiple disorders involving craving. Consistent with this idea, data suggest that manipulation of brain function (e.g., through neurostimulation of dorsolateral prefrontal cortex) may decrease food cravings like they do drug cravings (48).

Food cravings may be particularly relevant to individuals with obesity and eating disorders, and some interventions have targeted the management of food cravings. For example, food craving prior to food exposure has been linked to food consumption in obesity and to heightened levels in BED, raising the possibility that it has been targeted in treatment of the disorder (36). Notably, the Food and Drug Administration in the United States has recently approved a new medication combination of naltrexone and bupropion for the treatment of obesity. This follows several large studies reporting that the combination of these two medications, thought each to have some anti-craving effects, were effective in promoting weight loss in obese patients [e.g., Ref. (49, 50)]. However, to date, various other medications thought to reduce cravings have had limited effects on obese patients with BED (51–53). One study has found cognitive-behavioral therapy to be associated with better treatment outcomes and reduced food cravings in morbidly obese individuals undergoing bariatric surgery (54), and another study found that modifying a dialectical behavioral therapy by including appetite awareness and coping resulted in greater reductions in binge eating in patients with bulimia nervosa (55). Consistent with Buddhist views on craving described above, mindfulness-based approaches have shown promise with respect to reducing food cravings in some studies (56) and weight (57). However, other studies appear less promising (58), raising the possibility that there may exist individual differences with respect to who might respond favorably to these interventions [e.g., perhaps with respect to levels of food suppression thoughts (59) or susceptibility to the presence of food (60), with the possibility of gender-related differences also warranting consideration (61)]. The extent that behavioral techniques that target craving and methods of coping with craving are effective in the treatment of obesity and binge eating in different groups of individuals warrants additional investigation [e.g., (55)]. An alternate intervention, transcranial direct current stimulation of the prefrontal cortex, has been found in several studies to temporarily reduce craving (particularly in less impulsive individuals) and help them possibly resist food consumption (62, 63), although larger and more systematic studies are warranted to examine the clinical utility of this approach.

Food-craving states also warrant consideration within a developmental context. For example, upon food cue exposure in a group of children, adolescents and young adults, older age was associated with less craving, less recruitment of the striatum and greater recruitment of prefrontal cortex, and greater frontostriatal coupling (64). Adolescents have also shown less cortical activation in response to favorite-food cues as compared with adults (41, 65), with certain vulnerable groups of youth (for example, those with prenatal cocaine exposure) showing differences in striatal responses to favorite-food cues (66). The implications of these neurodevelopmental findings that examine responses to favorite-food cues and subjective craving responses on subsequent weight gain and the development (or not) of obesity or eating disorders remains to be more precisely elucidated.

In summary, food craving appears to be an important construct to consider, particularly within the current food environment. Approaches that might effectively target food cravings hold significant implications for advancing public health and clinical concerns relating to overeating.

## Conflict of Interest Statement

Dr. Potenza reports no conflicts of interest with respect to the content of this manuscript. He has received financial support or compensation for the following: Dr. Potenza has consulted for and advised Somaxon, Boehringer Ingelheim, Lundbeck, Ironwood, Shire, and INSYS; has received research support from the National Institutes of Health, Veteran’s Administration, Mohegan Sun Casino, the National Center for Responsible Gaming, and Forest Laboratories, Ortho-McNeil, Oy-Control/Biotie, Glaxo-SmithKline, and Psyadon pharmaceuticals; has participated in surveys, mailings, or telephone consultations related to drug addiction, impulse control disorders or other health topics; has consulted for law offices and the federal public defender’s office in issues related to impulse control disorders; provides clinical care in the Connecticut Department of Mental Health and Addiction Services Problem Gambling Services Program; has performed grant reviews for the National Institutes of Health and other agencies; has guest-edited journal sections and journals; has given academic lectures in grand rounds, CME events, and other clinical or scientific venues; and has generated books or book chapters for publishers of mental health texts. Dr. Grilo reports no conflicts of interest with respect to this manuscript. Dr. Grilo reports that he has received research support from the National Institutes of Health and Medical Research Foundations, has received honoraria for academic grand rounds and lectures at universities and professional conferences, has received honoraria for CME events and lectures, has received honoraria for academic journal editorial roles, has received consultant and advisory fees from Shire, and has received book royalties for academic books.

## Footnotes

^1^http://www.thefreedictionary.com/crave

^2^http://en.wikipedia.org/wiki/Ta%E1%B9%87h%C4%81
